# A Quality Improvement Project Aimed at Standardizing the Prescribing of Fluconazole Prophylaxis in a Level IV Neonatal Intensive Care Unit

**DOI:** 10.1097/pq9.0000000000000579

**Published:** 2022-07-18

**Authors:** Brandi Smith, Nipunie Rajapakse, Hannah E. Sauer, Kevin Ellsworth, Laura Dinnes, Theresa Madigan

**Affiliations:** From the * Department of Pharmacy, Mayo Clinic, Rochester, Minnesota; † Division of Pediatric Infectious Diseases, Department of Pediatric and Adolescent Medicine, Mayo Clinic, Rochester, Minnesota; ‡ Pharmacy Department, Texas Children’s Hospital, Houston, Texas; § Phoenix Children’s Hospital, Phoenix, Arizona.

**Keywords:** invasive candidiasis, fluconazole, neonate, neonatal intensive care unit, quality improvement

## Abstract

**Introduction:**

Invasive candidiasis has a high morbidity and mortality among premature neonates. Antifungal prophylaxis with fluconazole significantly lowers the risk of invasive fungal infection in this population. We noted the use of fluconazole prophylaxis in our level IV neonatal intensive care unit (NICU) was variable and sought to standardize prescribing of prophylactic fluconazole.

**Methods:**

We formed a multidisciplinary team to develop an evidence-based protocol using literature and expert consensus to guide appropriate use of fluconazole prophylaxis in our level IV NICU. After determining baseline fluconazole prophylaxis prescribing before protocol implementation, we used plan-do-study-act (PDSA) cycles to introduce protocolized prescribing and incorporate it into daily practice. A 6-month intervention phase was followed by a 2-year control phase, in which monthly audits were performed to evaluate protocol adherence. Results were displayed in a statistical process control chart.

**Results:**

Before protocol implementation, fluconazole prophylaxis prescribing adhered to the protocol in 81% of patients. During the first PDSA cycle, adherence increased significantly to 94.5% (86/91 patients), which further increased to 98.7% (74/75 patients) during the second PDSA cycle and remained at 96% (120/125 patients) during the control phase (*P* < 0.0001).

**Conclusions:**

A multidisciplinary group-designed protocol was successful in standardizing fluconazole prophylaxis prescribing for infants in the level IV NICU. Adherence to protocol was high following implementation and was sustained for the duration of the project. There were no cases of invasive candidiasis noted.

## INTRODUCTION

Invasive fungal infections occur in 2% to 4% of very low birth weight infants (<1500 g) and 4% to 16% of extremely low birth weight infants (<1000 g).^1^ This is due to a variety of factors including under-developed skin barriers, immunologic factors, abnormal microbial colonization of the gastrointestinal tract, and increased capability for gastrointestinal translocation. Additional factors further increase the risk of an invasive fungal infection, including central venous access, parenteral nutrition, broad spectrum antibiotics, and abdominal surgery, almost all of which a premature infant will encounter at some point during their neonatal intensive care unit (NICU) stay.^2,3^

Robust literature exists regarding the use of fluconazole prophylaxis in infants <1500 g to reduce the risk of invasive fungal infections.^3–10^ Numerous studies report decreased rates of colonization with *Candida*, decreased incidence of invasive fungal infections, and decreased mortality when fluconazole prophylaxis is used in these high-risk patients.^1,4,7,11–13^

Potential risks of fluconazole prophylaxis include selection for fluconazole-resistant *Candida* strains, drug related adverse effects, and drug-drug interactions, which can be amplified in a medically complex population. Consequently, prophylaxis use varies among institutions, is based on local epidemiology, and is generally reserved for the highest-risk population.

The Infectious Diseases Society of America (IDSA) recommends prophylaxis for invasive fungal infections with fluconazole for extremely low birth weight (<1000 g) infants admitted to a NICU with candidiasis rates higher than 10%, using a dose of 3–6 mg/kg/dose twice weekly for 6 weeks.^14^ The European Society of Clinical Microbiology and Infectious Diseases (ESCMID) recommends antifungal prophylaxis in neonates using risk stratification that includes invasive fungal infection rates, birth weight, and additional patient risk factors.^15^ Although these guidelines provide a framework for use, variability still exists between and within centers regarding when and how to use fluconazole prophylaxis in this vulnerable patient population.

The same practice variability existed in the level IV NICU at Mayo Clinic, which led to this quality improvement project. The goal of this project was to standardize fluconazole prophylaxis use by providing clear criteria for patient eligibility as well as recommendations for standard dose, route, and duration. This standardized approach was intended to ensure high-risk patients consistently received prophylaxis, while preventing unnecessary fluconazole exposure in low-risk patients who were unlikely to benefit from its use.

## METHODS

### Context

This quality improvement project was conducted in the level IV NICU of the Mayo Clinic Children’s Center, an academic tertiary care center in Rochester, Minnesota. This 148-bed pediatric hospital stands within the adult hospital and includes a 35-bed level IV NICU. Forty-four percent of neonates cared for in the NICU are inborn; however, the level IV NICU is also a regional referral center that accepts transfers from the surrounding area. Patients admitted to the NICU include preterm infants (≥22 weeks’ gestation), infants with congenital anomalies, including complex surgical and cardiac conditions, and term infants with respiratory or other medical conditions. Over 350 infants receive care in the level IV NICU annually, of which 50% to 60% are preterm. As the level IV NICU admits patients at the highest risk for neonatal invasive candidiasis, this unit was chosen for implementation of this quality project.

### Planning the Intervention

In 2017, a wide range of prescribing practices for fluconazole prophylaxis existed in our level IV NICU. This included variations in patient eligibility, medication dosing, route, and duration. This raised concerns for medication misuse, medication-related adverse effects, the potential for preventable cases of invasive candidiasis due to prophylaxis under-utilization, and the development of fluconazole-resistant *Candida* strains due to potential prophylaxis overuse. With the identification of this quality gap, we recognized the need for a standardized approach to fluconazole prophylaxis prescribing. We assembled a project team including representatives from pediatric infectious diseases, neonatology, and pediatric pharmacy. This team employed the Six Sigma tool of Define, Measure, Analyze, Improve, Control (DMAIC) to guide the project from start to completion. The primary aim of this project was to increase protocol-concordant use of fluconazole prophylaxis to >90% over a 6-month study period.

### Defining the Intervention

The define phase of this project sought to understand the quality gap in practice variability. A survey was developed and distributed to the neonatologists caring for patients in the level IV NICU to assess baseline prescribing practices and gain insight into perceived patient eligibility, variation in fluconazole dose prescribed, and duration of prophylaxis deemed necessary.

**Fig. 1.**
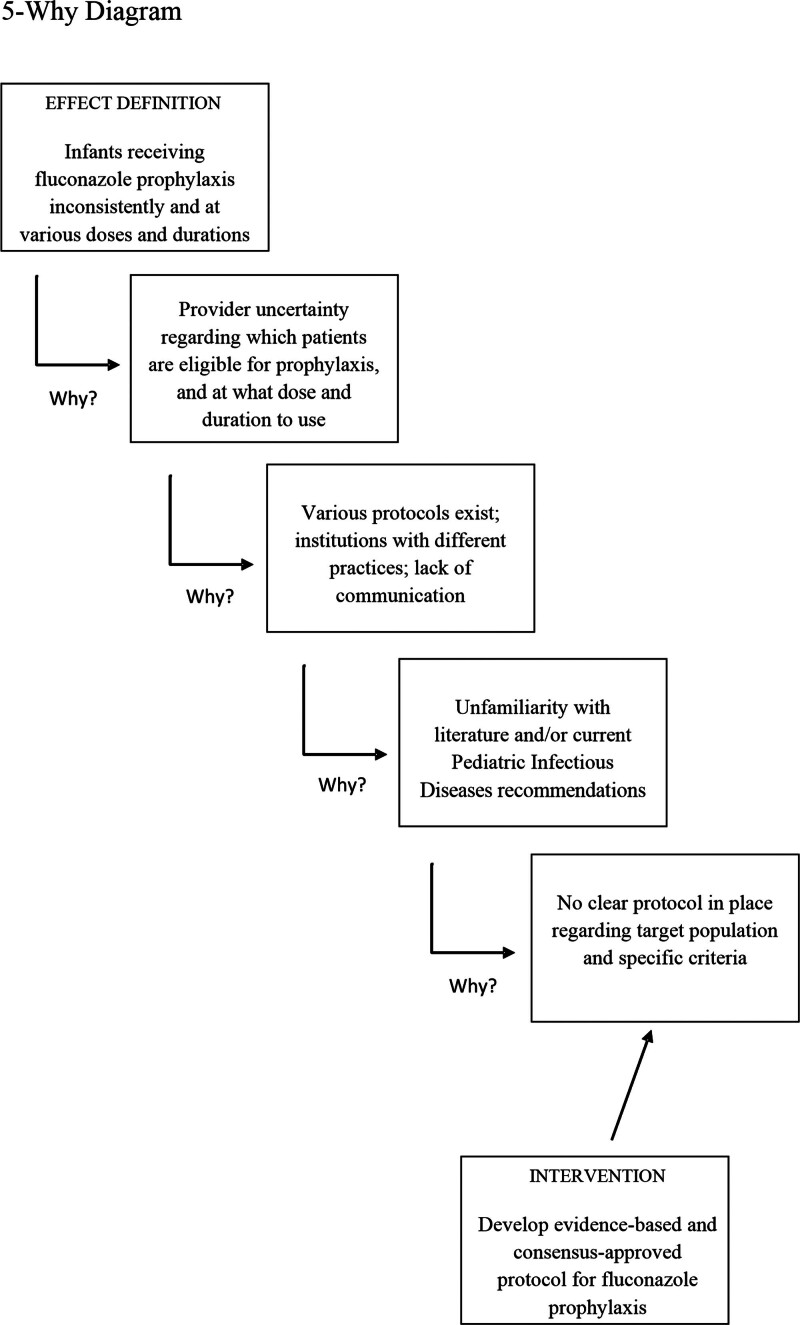
5-Why Diagram analysis of causes of fluconazole prophylaxis prescribing variability in the NICU.

**Fig. 2.**
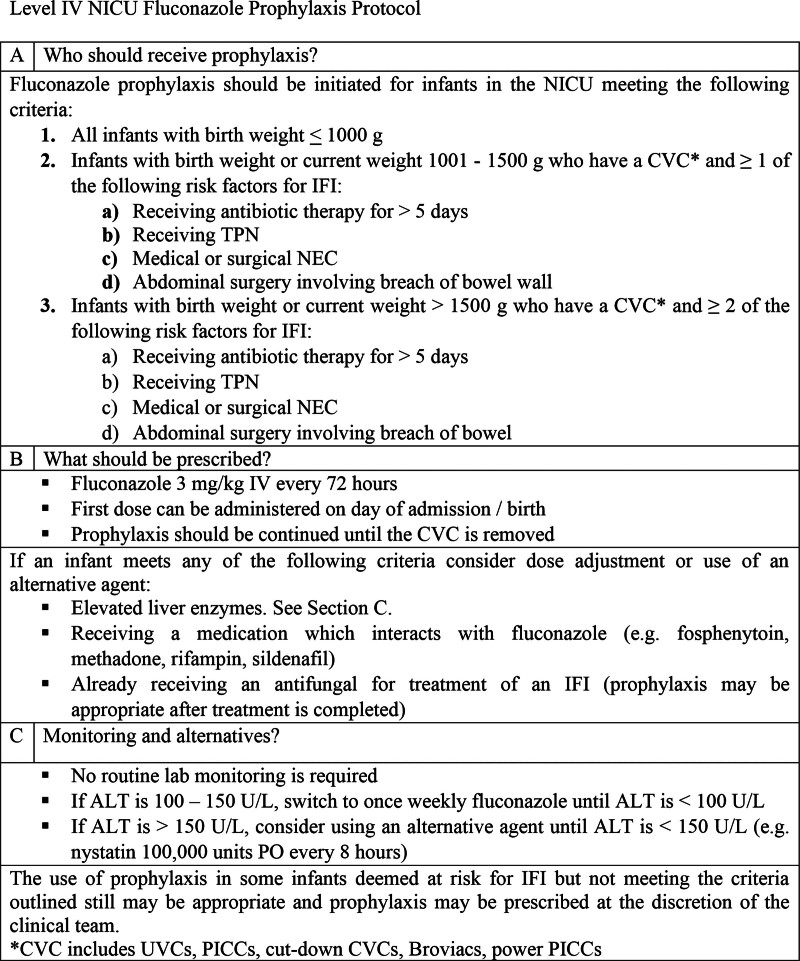
Evidence-based and expert consensus protocol for use of fluconazole prophylaxis for invasive candidiasis implemented in the Level IV NICU. ALT, alanine aminotransferase; CVC, central venous catheter; IFI, invasive fungal infection; NEC, necrotizing enterocolitis; PO, by mouth; TPN, total parenteral nutrition.

The survey results were incorporated into a 5-Why Diagram to identify the root causes of prescribing variabilities and recognize areas where fluconazole prophylaxis could be standardized (Fig. 1). Analysis identified two prescribing variation root causes: uncertainty regarding which patients should receive fluconazole prophylaxis, and dose and duration of use. Therefore, the primary intervention targeted the development of a protocol to address these uncertainties. The quality team developed a simple, evidence-based protocol for use in the level IV NICU through a literature review, survey of fluconazole prophylaxis practices at other level IV NICUs around the country, and expert consensus (Fig. 2). Using the information gathered, the protocol defined high-risk patients eligible for prophylaxis using the following criteria: weight, risk factor(s) for invasive candidiasis, and presence of a central line. The protocol recommended a standard prophylaxis dose of 3 mg/kg given IV every 72 hours for the duration of central line placement.

After the protocol was created, it was distributed to neonatologists, pediatric infectious diseases physicians, and pediatric pharmacists with a description of the quality project. Feedback from the stakeholders was incorporated into a revised version of the protocol, which was presented to the NICU Quality and Safety Committee for approval. The finalized one-page protocol was emailed to all stakeholders with education describing their role in the project. The protocol was also placed in the level IV NICU workroom for reference and uploaded onto the internal NICU resource website for easy access.

### Intervention

The improve phase of the project spanned a six-month period, from January 1, 2018, through June 30, 2018, and consisted of two serial plan-do-study-act (PDSA) cycles.

#### PDSA Cycle 1: Protocol Introduction (January–March 2018)

The protocol was integrated into clinical practice with the goal of increasing capture of eligible patients and concordant prescribing with the protocol. The NICU service incorporated its review into their daily rounding template, prompting the resident or neonatal nurse practitioner caring for the patient to address fluconazole prophylaxis need when discussing infectious diseases concerns. In addition, a daily review to assess whether a patient met eligibility criteria for starting, continuing, or discontinuing prophylaxis was incorporated into the workflow of the pharmacists monitoring the NICU.

#### PDSA Cycle 2: Quality Checklist Implementation (April–June 2018)

The results of cycle 1 were reviewed. Ongoing compliance concerns were noted because ordering relied on the provider to remember to discuss the protocol during rounds. This obstacle was reviewed by the quality team and addressed at a NICU Quality and Safety Committee meeting. Ultimately, this led to the recruitment of the NICU medical assistant for additional support. The medical assistant maintained a NICU quality checklist database, which was updated daily with information about various unit quality practices and chronic disease monitoring. The quality team asked the assistant to add fluconazole eligibility criteria to the database as an additional means of review. After daily updates were made, the assistant and neonatal medicine fellow on service reviewed the data to identify patients deviating from protocol. For those identified patients, the neonatal fellow discussed the patient with the resident or nurse practitioner to assess whether there was a clinical need for the deviation. If no clinical need was identified, the patient was reconciled to protocol.

### Study of the Intervention

To measure the impact of a protocolized approach to prescribing fluconazole prophylaxis, a retrospective chart review of 100 consecutively admitted infants between February and July 2017 was performed to capture baseline prescribing practices. A 6-month washout period followed and the quality improvement project began in January 2018. Two 3-month long PDSA cycles of prospective audits of all admitted infants were conducted from January–March 2018 and April–June 2018 by chart review.

To ensure gains made during the improvement phase were maintained, a postimplementation control phase was conducted in accordance with Mayo Clinic’s Quality Academy requirements. We performed regular monitoring of the quality project’s primary aim through review of fifteen patients per quarter for a 2-year time period. Protocol compliance was assessed by random selection of five NICU patients every month to ensure real-time monitoring and feedback and facilitate intervention if needed. In total, we monitored 125 NICU patients during the control phase.

For the two PDSA cycles and postimplementation control cycle, patient data were monitored weekly until NICU discharge to ensure changing eligibility criteria were captured and fluconazole prophylaxis was evaluated accordingly (eg, appropriately discontinued at time of central line removal). For infants that met criteria for prophylaxis on more than one occasion during their NICU stay, only the first course was included for the purpose of this project.

### Measures

The primary outcome measure was the percent of patients prescribed fluconazole prophylaxis according to protocol among all patients admitted to the NICU during the project timeframe. The balancing measure was the rate of invasive candidiasis for all patients in the level IV NICU during the project timeframe, as assessed through chart review of all NICU patients during the intervention phase, as well as surveillance records kept by Mayo Clinic’s Infection Prevention and Control team. Additionally, evaluation for *Candida* positive blood cultures for all patients in the NICU during the project timeframe was performed using records obtained by Mayo Clinic’s bacteriology laboratory.

Baseline aminotransferase levels were not routinely monitored; however, the protocol provided guidance on how to adjust prophylaxis in the event of elevation. Dose adjustment in the setting of renal impairment was considered; however, given the low dose of 3 mg/kg IV every 72 hours, no adjustment was made.

### Analysis

**Fig. 3.**
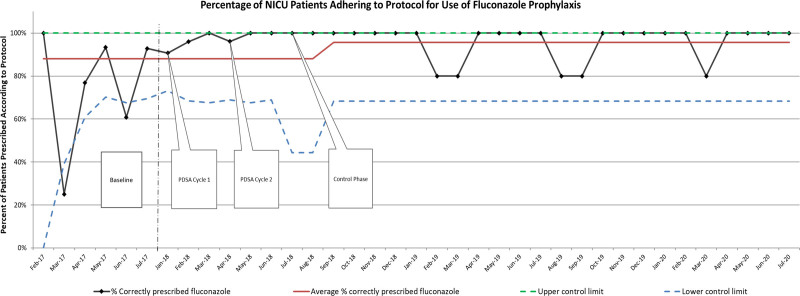
P-chart depicting the monthly percent of NICU patients adherent to fluconazole prophylaxis protocol. Center line shifts are determined by standard statistical process control chart rules for special cause center line shifts.

All data were collected and analyzed through Microsoft Excel (Microsoft Corporation, Redmond, VA) and JMP Software (SAS Institute, Inc, Cary, NC). A statistical process control p-chart (Fig. 3) was developed using Microsoft Excel to track the primary outcome variable (percent of patients prescribed according to protocol). Center line shifts were determined using the 8 consecutive points above or below the previous center line as described by Provost and Murray.^16^ A *P* value <0.05 was considered statistically significant.

### Ethical Considerations

This project was deemed a quality improvement project and not human subjects research by the Mayo Clinic Institutional Review Board and was therefore exempt from review.

## RESULTS

Baseline fluconazole prophylaxis use in accordance with the protocol before implementation was 81% (n = 81/100). For the nineteen patients in which fluconazole use did not match protocol, the following reasons were noted (more than one apply in some cases): prophylaxis was not given when indicated (n = 5), the dose of fluconazole differed from protocol (n = 14), prophylaxis was given when not indicated (n = 6), and prophylaxis was not stopped when no longer needed (n = 1). Adherence increased to 94.5% (n = 86/91) in the first PDSA cycle, and 98.7% (n = 74/75) in the second PDSA cycle. For the six patients in which fluconazole use did not match protocol during the two PDSA cycles, the following reasons were noted: prophylaxis was not given when indicated (n = 1), prophylaxis was given when not indicated (n = 4), prophylaxis was not stopped when no longer needed (n = 1). Overall, adherence increased to 96.4% in the 6 months following protocol introduction (n = 160/166 versus 81/100 at baseline, *P* < 0.0001). In total, 16 infants (16%) in the baseline group and 47 (28%) in the intervention group received fluconazole prophylaxis. There were no cases of invasive candidiasis or *Candida* positive blood cultures in the baseline or intervention groups.

In the 2-year control phase, protocol adherence was 96% with 36 infants (29%) receiving fluconazole prophylaxis. There were no cases of invasive candidiasis or *Candida* positive blood cultures reported in the audited or nonaudited patients.

The primary aim of >90% protocol-concordant prescribing of fluconazole prophylaxis was achieved with completion of PDSA cycle 2. As noted in the p-chart (Fig. 3), special cause variation was achieved shortly after onset of the control phase, which was sustained for the duration of the project timeframe.

## DISCUSSION

We demonstrate that through quality improvement efforts, implementation of protocolized prescribing for the use of fluconazole prophylaxis can effectively standardize prescribing and limit use to the highest-risk population. Restricting the use of prophylaxis to a circumscribed group did not result in increased rates of candidiasis in our NICU, which was a concern surrounding this project. Like other authors, we also did not see emergence of fluconazole-resistant *Candida* strains during the project timeframe, as would be a concern with overuse of fluconazole.^13,17^ The protocol developed through this quality improvement project remains standard practice in our level IV NICU at the time of writing this manuscript.

Application of the Model for Understanding Success in Quality (MUSIQ) framework identified several factors that contributed to the overall success of this quality improvement project.^18^

At an organizational level, Mayo Clinic is an avid supporter of quality improvement efforts. Employees are encouraged to take part in quality initiatives and are offered a variety of training opportunities through the Mayo Clinic Quality Academy. Additionally, quality improvement team factors included content expertise from infectious diseases, neonatology, and pharmacy which helped create a solid foundation for this project.

At a microsystem level, our NICU embraces quality improvement efforts and has seen many successful quality projects implemented in recent years. Consequently, the NICU is familiar with the quality improvement processes used in this project. Additionally, the NICU is accustomed to protocolized guidelines for patient care and medication use; therefore, this protocol was easily integrated into a practice already primed for use. Furthermore, there was motivation from physicians and pharmacists to standardize an approach to fluconazole prophylaxis to reduce prescribing variability. Providing a protocol outlining qualifying criteria and fluconazole dosing recommendations gave prescribers clear direction with little time or effort required. These factors likely contributed to the significant improvement in protocol-concordant fluconazole use in PDSA cycle 1.

During PDSA cycle 2, protocol noncompliance was addressed through daily review of patient eligibility using the quality checklist. This additional layer of accountability further increased adherence to protocol. As the project progressed and real-time data showed protocol use did not result in increased rates of candidiasis, stakeholder confidence in the quality project increased, which further solidified its place in the NICU practice.

Five of the 125 patients audited during the control phase did not receive fluconazole prophylaxis despite meeting eligibility criteria. Allthough many factors may have contributed to this, the neonatal medicine fellow and medical assistant, both of whom had shared the responsibility of daily review for eligibility, had moved on from their positions at the time the control phase began. It is likely that dissolution of the daily review contributed to these omissions. Following the conclusion of this quality project, the fluconazole prophylaxis protocol was incorporated into a neonatal admission order set in the electronic medical record to offset the potential for future omissions.

### Limitations

Given the ongoing use of fluconazole prophylaxis before protocol implementation, true baseline rates of invasive candidiasis in our NICU in the absence of prophylaxis could not be determined. This presents a significant limitation to the project, for without baseline candidiasis rates it is unknown whether protocol-eligible patients continue to be at high risk of invasive candidiasis. As a result, this creates the potential for overuse of fluconazole in this vulnerable population.

Another limitation of this project is the single-center design. The strategies used to implement this protocol successfully may not be directly transferable to other institutions. In addition, the epidemiology of fungal infections and patient populations may differ at other centers, and therefore, chosen criteria for the use of fluconazole prophylaxis may be different. Although this protocol could be adapted to other NICUs, it may require modification and individualization to ensure similar success.

Finally, we chose a 6-month washout period between the collection of baseline data and data associated with the project interventions. This was done to omit any baseline prescribing changes that may have been influenced by knowledge of the upcoming quality improvement project. In choosing to omit this period of time, it is possible that the baseline data does not accurately represent true prescribing practices of fluconazole prophylaxis before protocol implementation.

## CONCLUSION

This quality improvement project aimed to improve patient safety and outcomes by encouraging reliable and consistent fluconazole prophylaxis use in high-risk infants while avoiding use in low-risk infants, all while not increasing invasive candidiasis rates.

Implementation of an evidence-based protocol using a multidisciplinary approach led to standardization of fluconazole prophylaxis for invasive candidiasis in our level IV NICU. By addressing the root causes of variability behind prescribing practices, this quality improvement project standardized the approach and provided clear guidelines for prescribing. There were no increased rates of invasive candidiasis after adoption of this protocol and no infections with fluconazole-resistant *Candida* strains.

## ACKNOWLEDGMENTS

Assistance with the study: We wish to thank Dr. Stephanie Mavis for her valued insight in revision of this article.

## DISCLOSURE

The authors have no financial interest to declare in relation to the content of this article.
